# Higher Oxidative Stress in Endometriotic Lesions Upregulates Senescence-Associated p16^ink4a^ and β-Galactosidase in Stromal Cells

**DOI:** 10.3390/ijms24020914

**Published:** 2023-01-04

**Authors:** Helena Malvezzi, Bruna Azevedo Cestari, Juliana Meola, Sérgio Podgaec

**Affiliations:** 1 Hospital Israelita Albert Einstein, Sao Paulo 05652-900, Brazil; 2 School of Medicine of Ribeirao Preto, University of São Paulo, Gynecology and Obstetrics, Av. Bandeirantes, 3900, Vila Monte Alegre, Ribeirão Preto 14049-900, Brazil

**Keywords:** endometrium, endometriosis, eutopic, reactive oxygen species, oxidative stress, MAPK

## Abstract

Endometriosis affects a significant proportion of women worldwide; however, no definitive cure for this disease has been discovered to date. Oxidative stress promotes endometriotic lesion maintenance in the peritoneal cavity in women. Furthermore, there is evidence of the mitogen-activated protein kinase (MAPK) signaling pathway and senescence involvement in the physiopathogenesis of endometriosis. Reactive oxygen species (ROS) cause oxidative damage and are expected to trigger senescence in the endometrium while also causing alterations in MAPK signaling. However, the role of ROS in the senescence-associated phenotype in endometriosis remains unknown. In this context, this study attempted to delineate the pathways linking ROS to senescence in endometrial and endometriotic lesions of healthy individuals and those with endometriosis. Our results indicate a higher presence of ROS in endometriotic lesions, and the upregulation of MAPK. Furthermore, we show that endometriotic lesions in stromal cells stimulated with hydrogen peroxide develop more senescence traits than eutopic and non-endometriosis endometrium. Overall, endometriotic cells respond differently to extracellular distress. Our contribution to further research in this field contributed to the roadmap of endometriosis’ search for alternative treatments.

## 1. Introduction

Oxidative stress (OS), characterized as an imbalance in the redox system between the production and neutralization of reactive oxygen species (ROS), appears to play an important role in the peritoneal cavity of women with endometriosis [1,2]. OS leads to macromolecular oxidative and tissue damage, and chronic inflammation, and regulates the expression of genes encoding immune regulators, cytokines, and cell-adhesion molecules [3,4]. At the macromolecular level, OS promotes angiogenesis and vascularization, adhesion, proliferation, and cellular invasion of endometriotic lesions in the peritoneal cavity [5]. Damage has been observed in proteins, lipids, and cell membranes, leading to DNA fragmentation and the perturbation of the cell cycle [6,7,8], indicating a link between OS, the mitogen-activated protein kinase (MAPK) pathway, apoptosis, and senescence [9,10].

There is evidence of the involvement of the MAPK signaling pathway [11] and senescence [12,13,14] in the physiopathogenesis of endometriosis. The extracellular signal-regulated protein kinase (ERK)-mediated pathway modifies cell growth, proliferation, and survival [15,16]. MAPK p38 upregulates inflammation, cell differentiation, growth, and cell death [17]. Furthermore, the c-Jun N-terminal kinase (JNK)-mediated pathway, which is a cellular-stress-activation pathway, controls cell proliferation and apoptosis [18]. Similarly, senescence markers present in the endometriotic lesion [13] can alter tissue homeostasis, contributing to the maintenance of lesions and the characteristic inflammatory milieu of the disease. Previous studies have linked the increase in endometrial stromal cells’ senescence to implantation failure and impaired decidualization [19,20], but no association with endometriosis or endometriosis-associated infertility is known.

The connection between OS and excessive ROS with endometriosis and senescence is based on the ability of ROS to cause oxidative damage in cells, which triggers the senescent state [21]. It can also alter the major MAPK proteins, elicit inflammation, change the cellular fate, and transform the phenotype of the damaged cells into a long-lasting senescence-associated secretor phenotype (SASP) [22]. OS is associated with oocyte aging because it is responsible for meiotic distress through spindle obliteration and telomere shortening [23]. Furthermore, cumulus granulosa cells from patients with endometriosis were triggered to senescence by excessive ROS [24], which could be related to endometriosis-associated infertility.

Although the involvement of ROS in tissue damage in endometriosis is understood, its role in the endometriosis senescence-associated phenotype remains unclear. Considering what is known regarding the physiopathogenesis of endometriosis, and the importance of OS in the development of the disease and activation of inflammatory and senescence pathways, the relationship between the regulation of ROS and senescent markers in endometriosis requires further investigation. We hypothesized that ROS are involved in the upregulation of senescence markers. Hence, we aimed to investigate the presence of OS biomarkers and alterations in the MAPK-signaling pathway in endometriosis, to further stimulate stromal cells with hydrogen peroxide (H_2_O_2_) and to observe the cellular response using senescence markers (p16^ink4^ and lamin B1). The results demonstrated in this study contribute to the understanding of cell fate in endometriotic lesions, especially senescence, and provide insights into the long-term persistence of the disease. 

## 2. Results

### 2.1. Higher Oxidative Damage Was observed in Endometriotic Lesions Than the Eutopic Endometrium

To evaluate oxidative stress (OS) impact in endometriosis and cellular senescence, indirect methods should be accomplished to transform OS into a tangible measure. Since reactive oxygen species are unstable molecules with short half live, indirect measures are used. For instance, the FOX system detects hydroperoxides formation, MDA is a secondary product of lipidic peroxidation and carbonyl is one of the main protein oxidation markers. 

**Figure 1:**
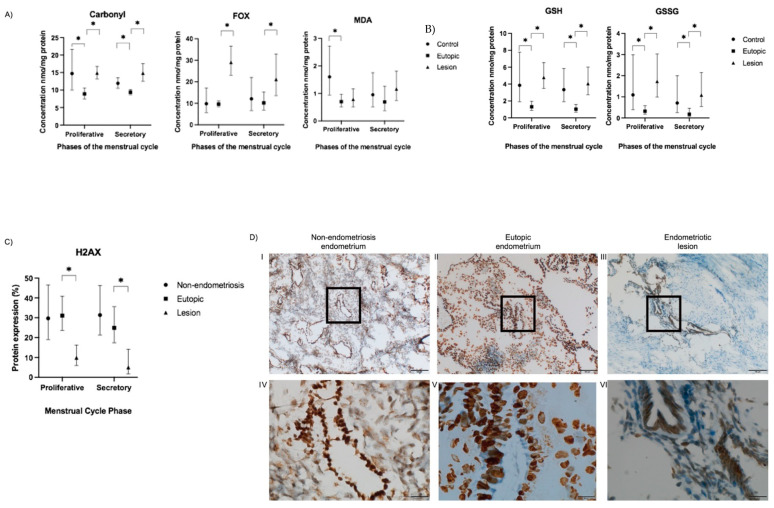
Oxidative stress markers (FOX, MDA, carbonyl, GSH, GSSG, and H2A.X) in tissue samples (lesion, eutopic endometrium, and non-endometriosis control) and in different menstrual cycle phases (secretory and proliferative phase) of patients with and without endometriosis. Data are expressed as estimated mean values and 95% confidence intervals. Concentration of (**A**) carbonyl; FOX; MDA; (**B**) GSH; GSSG; and (**C**) H2A.X. (**D**) Images of H2A.X protein labeling in endometriosis tissues and controls. Brown represents staining by the H2A.X antibody, and blue marks the cell nuclei; the blank spaces were not considered in the quantitative analyses. Scale bar: (**I**–**III**) 100 µm; (**IV**–**VI**) 20 µm. * *p* < 0.05. MDA: malondialdehyde; FOX: ferric-orange xylenol, GSH: reduced glutathione; GSSG: oxidized glutathione; and H2A.X: histone H2A variant.

The mean value of FOX in the endometriotic lesions was 2.15-fold higher than that in eutopic tissue (*p* = 0.005) in the secretory phase and 2.99-fold higher (*p* = 0.001) in the proliferative phase. Lesions in the secretory phase had 66% (*p* < 0.001) more carbonyl groups than that of the eutopic tissue. In the proliferative phase, the lesions’ carbonyl concentration was 64% (*p* < 0.001) higher than that in the eutopic tissue in the proliferative phase, while no difference in MDA level was observed (Figure 1A). The lesions had high levels of GSH and GSSG in both phases of the menstrual cycle (Figure 1B). 

In contrast, we found that the average amount of carbonyls in the non-endometriosis group was 28% (*p* = 0.032) higher in the secretory phase and 99% higher (*p* < 0.001) in the proliferative phase than that of the eutopic endometrium group. The level of MDA in the control group was 2.41 times (*p* = 0.030) higher than that in the eutopic tissue in the secretory phase (Figure 1A), and the control group also had higher GSH and GSSG (Figure 1B). 

### 2.2. DNA Damage Was Not Observed in Endometriotic Lesions but Was Present in Eutopic Endometrium

Senescence-associated DNA damage can be measured by its products, 8OHdG and H2A.X. 8OHdG analysis was performed on 22 samples. Of these, nine were eutopic endometrium, 13 non-endometriotic tissue, and 13 endometriotic lesions. Differences in 8OHdG level were not observed among the groups. We evaluated H2A.X protein expression in eutopic endometrium and endometriotic lesions tissues of patients with or without endometriosis in immunostained tissue sections. Representative images of H2A.X immunoreactivities are shown in Figure 1D. H2A.X was detected in all samples. There was uniform, strong, and higher nuclear H2A.X expression in epithelial and stromal cells from eutopic endometrium than from endometriotic lesions (*p* < 0.001) (Figure 1C,D). In endometriotic lesions, H2A.X expression was absent or faint in nuclei of stromal cells (Figure 1D), whereas epithelial cells were H2A.X positive. Non-endometriosis endometrium H2A.X expression was increased in both epithelial and stromal cells. 

### 2.3. Endometriotic Lesions Displayed Upregulation of MAPK Pathway Proteins

Recent studies have increasingly demonstrated the importance of the MAPK pathway in endometriosis [17]. Its main kinases are deregulated, demonstrating a central role in the regulation of the immune response, and its relationship with one of the main symptoms, pelvic pain, demonstrates that the inhibition of ERK1/2 reduces the expression of P2X3, a sensory receptor [11]. 

**Figure 2:**
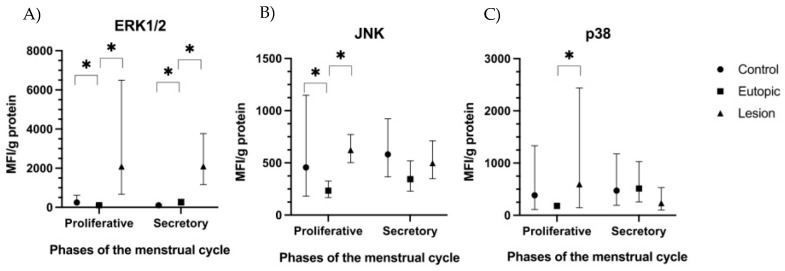
Concentrations of MAPK pathway proteins in tissue samples (lesion, eutopic endometrium, and non-endometriosis control) from patients with and without endometriosis (secretory and proliferative phases). Data are expressed as estimated mean values and 95% confidence intervals. (**A**) ERK1/2; (**B**) JNK; and (**C**) P38. * *p* < 0.05. ERK: extracellular signal-regulated kinases; JNK: c-Jun NH2-terminal kinase.

The levels of the three main phosphorylated MAPK proteins were higher in the endometriotic lesions than in the eutopic endometrium in the proliferative phase. The phosphorylated ERK1/2 level was higher in both phases of the menstrual cycle (9-fold in the secretory phase and 29.57-fold in the proliferative phase; *p* < 0.001), while the phosphorylated JNK (2.62-fold; *p* = 0.004) and phosphorylated p38 (5.17-fold; *p* = 0.016) levels were higher in the proliferative phase. Non-endometriosis samples had higher ERK1/2 (0.42-fold; *p* = 0.038 in the secretory phase, and 2.98-fold; *p* = 0.026 in the proliferative phase) and JNK (2.94-fold; *p* = 0.002 in proliferative phase) than the eutopic endometrium in the same phase (Figure 2). 

### 2.4. Free Radicals Upregulated β-Galactosidase and p16^ink4^ and Deplete Lamin B1 in Stromal Cells

Cellular senescence is characterized by the state of permanent blockage of the cell cycle caused by intrinsic and extrinsic stimuli of the cell. The protein p16^Ink4a^ is a regulator of the cell cycle, and lamin b1 depletion has been associated with senescence. Although H_2_O_2_ is not a free radical, it is considered a ROS because of its damage potential; thus, it is used as an OS stimuli. 

**Figure 3:**
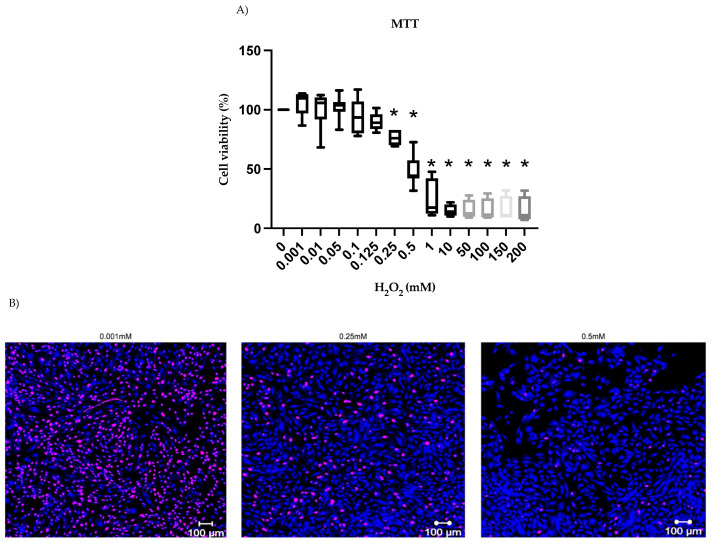
Hydrogen peroxide concentration assay. Stromal cells from endometriotic lesion, eutopic endometrium, and non-endometriosis endometrium were used for the H_2_O_2_ assay. (**A**) MTT cell viability test; (**B**) Ki-67 staining in stromal cells. Blue represents cell nuclei, and pink indicates the cells marked for the Ki-67 antigen. * *p* < 0.05.

**Figure 4:**
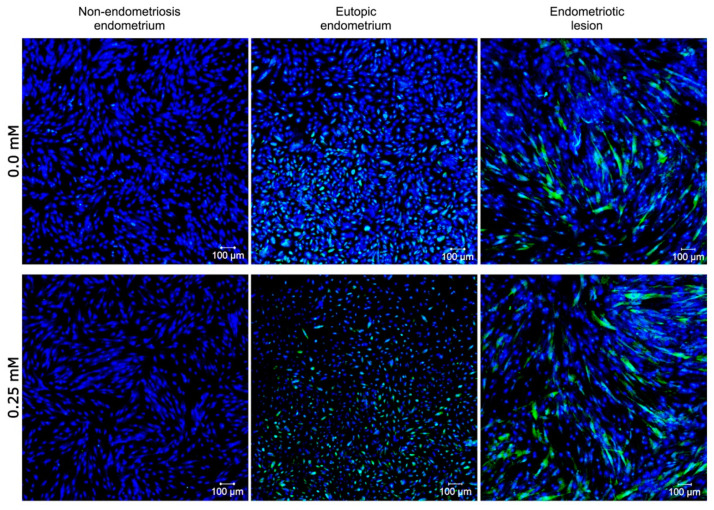
p16^ink4^ expression in stromal cells from endometriosis and non-endometriosis endometrium. Fluorescence intensity of p16^ink4^ (in green) in the stromal cells from non-endometriosis and endometriosis endometrium and endometriotic lesions at passage 6 stimulated with 0.0 mM and 0.25 mM H_2_O_2_, analyzed using indirect immunofluorescence. Nuclei were stained with DAPI (in blue).

After nine repetitions, concentration assays suggested 0.25 mM to be the ideal concentration for H_2_O_2_ stimuli as there was a significant drop (*p* < 0.05) in cell viability (Figure 3A) and proliferation (Figure 3B). H_2_O_2_-treated stromal cells (endometriotic lesion, eutopic endometrium, and non-endometriosis tissue) increased the relative expression of p16^Ink4a^ (Figure 4). The ratio in 79% of the samples (11/14) was higher than 1 (Supplementary Table S1), indicating that p16^Ink4a^ expression was greater in the group of cells stimulated with 0.25 mM H_2_O_2_ than in cells cultured without H_2_O_2_ (0.0 mM). This difference was noticed in cells from all tissue types (endometriotic lesion, eutopic endometrium, and non-endometriosis tissue).

**Figure 5:**
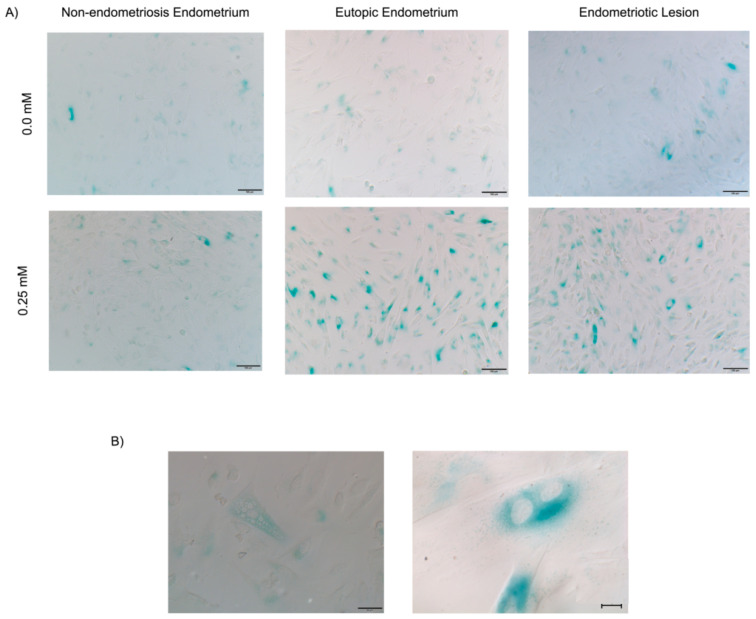
Senescence-associated β-galactosidase assay. Senescence-associated β-galactosidase intensity (in blue) in the stromal cells from non-endometriosis and endometriosis endometrium and endometriotic lesion at passage 6 stimulated with (**A**) 0.0 mM and 0.25 mM H_2_O_2_ and analyzed using indirect immunofluorescence; scale bar: 100 µm (**B**) multinucleation in stromal cells from the endometriotic lesion; scale bar 20 µm.

Lamin B1 expression varied among the groups (Supplementary Table S1); however, endometriotic lesions presented depleted lamin B1 in three out of four experiments, and Lamin B1 in eutopic endometrium stromal cell was upregulated in almost all samples (five out of six) after H_2_O_2_-treatment. Furthermore, β-galactosidase was detected in all endometriotic lesions (in the presence of 0.0 mM and 25 mM H_2_O_2_) and the eutopic endometrium (Figure 5A) but was absent or negligible in the non-endometriosis group. We also observed the multinucleation and morphological alteration (Supplementary Figure S1) characteristics of senescence in cells stimulated with H_2_O_2_ and labeled with β-galactosidase (Figure 5B).

## 3. Discussion

We aimed to identify the profile of kinase signaling pathway proteins (ERK, JNK, p38) in the endometrium and endometriotic lesions and verify the impact of OS (FOX, MDA, carbonyl, 8OHdG, H2A.X, GSH, and GSSG), quantified in endometriotic tissue, on stromal cell senescence makers (p16^Ink4a^, lamin B1, and SA-β-gal). The characteristics of the endometriotic lesions indicate the presence of consistent oxidative stress and the dysregulation of MAPK expression, and the deleterious impact of ROS on the expression of senescence markers was observed in stromal cells from endometriotic tissues. 

ROS can damage lipids and proteins and consequently alter levels of cellular membrane fluidity, leading to changes in the structure, permeability, and antigenicity of cells [25,26,27], which could be associated with the dysfunctional immune system detected in patients with endometriosis [28,29]. Furthermore, modifications in membrane fluidity affect active and passive transport, enzymatic functions, hormone-mediated signal transduction and regulation, proliferation, cell cycle, and aging [30,31]. Depleted membrane fluidity was detected in oxidative stress-induced senescence-susceptible animals [32]. On the one hand, the physiological level of cellular ROS function as redox messengers can be used for intracellular signaling. On the other hand, ROS accumulation stimulates downstream redox-sensitive signaling pathways, such as MAPK, to modify the cellular fate and can directly modulate the activation of key signaling molecules, as well as induce cell senescence in the aging process [33]. Furthermore, ROS accumulation in IDH1 (isocitrate dehydrogenase-1—a potent antioxidant) knockdown ovarian granulosa-like tumor cell line triggered the phosphorylation of ERK ½, JNK, and p38 and accelerated senescence, suggesting that ROS was responsible for cell cycle arrest via the MAPK signaling pathway [34].

In this study, throughout the menstrual cycle, higher levels of ROS (carbonyl and FOX) were detected in the endometriotic lesions, indicating a persistent redox disequilibrium. Although higher levels of ROS markers have been systematically detected in endometriosis [5,35], only a few studies have directly investigated their levels in tissue [36], and most of them have been reported in primary cultivated stromal cells [37,38] or ovarian tissue [39,40]. The deleterious presence of ROS in endometriotic lesions is likely to contribute to the dysregulation of signaling pathways, alterations in gene and protein expression, and metabolic dysfunction, including senescence traits [41,42]. 

As discussed above, signaling pathways, such as the MAPK pathway, are susceptible to redox modeling [43]. Senescence-inducible proteins are upregulated due to sensitization of the MAPK pathway by ROS, leading cells to exhibit long-term growth arrest and SASP. ERK1/2, JNK, and p38 are the three main classes of MAPKs that contribute to senescence traits [44]. Along with the persistent ROS accumulation in endometriotic lesions, we detected the upregulation of the three main MAPKs in the proliferative phase, and ERK1/2 upregulation was also observed in the secretory phase. Our data are in line with other studies where the involvement of ERK1/2 and p38 pathway in the pathogenesis of endometriosis was demonstrated [9,15,16]. To date, the majority of the current data present in vitro MAPK evaluation; previous studies have described the same effect in endometriotic stromal cells [45] and peritoneal fluid [46], which confirms our findings. Furthermore, a *GWAS* analysis considered the MAPK pathway, especially ERK1/2, as the most significant pathway associated with endometriosis, possibly reflecting a causal mechanism between altered MAPK expression and the development of endometriosis [47]. The results of the analysis of the post-translational modifications of the main MAPK proteins were in agreement with the results of the transcriptional analysis. The expression of MAPKs was altered from gene to protein levels in the endometriotic lesions. Additionally, the findings of Borodkina and colleagues [48,49] demonstrated that endometrial stem cells exposed to sublethal oxidative stress undergo premature senescence and p38 activation. They analyzed the effect of p38 inhibition on the H_2_O_2_-induced premature senescence of endometrial stem cells, and the results show partial proliferation recovery, cell size maintenance, and an increase in pRB, demonstrating both the importance of p38 in cellular senescence and of H_2_O_2_ triggering senescence through p38 activation.

MAPK pathways initiate the senescence cascade [50] and upregulate proteins such as p16^inka4^, which are responsible for maintaining the growth arrest state indefinitely. Previously, we detected a higher expression of p16^inka4^ in endometriotic lesions compared to that in the eutopic endometrium [13]. The upregulation of both MAPKs and p16^inka4^ describe a scenario favorable for the establishment of senescence traits in endometriotic lesions. Notably, SA-β-gal activity and the upregulation of the classical senescence-associated gene, p16^INK4A^, and heterochromatin foci, all hallmarks of senescence [51,52], correlate with an increase in the ERK pathway activity [53]. 

After stimulating the endometriotic lesions and eutopic endometrium stromal cells with a non-lethal dose of H_2_O_2_, we observed an increase in SA-β-gal activity and the upregulation of p16^INK4A^ at the same time that a sutil downregulation of Lamin B1 was noticed in endometriotic lesion stromal cell. In addition, binucleation and flattened morphology were also observed in ROS-stimulated cells, which are also features of senescent cells [54]. The current results suggest a correlation between the increase in ROS levels and senescence traits in endometriosis; previous studies have shown the correlation between ROS and senescence in other cell type and disease [49,55]. Moreover, stromal cells from endometriotic lesions showed the most pronounced changes before and after H_2_O_2_ stimulation. Stromal cells from patients without endometriosis showed minor changes. Interestingly, mesenchymal cells isolated from the eutopic endometrium of non-receptive patients presented higher senescent markers (p21, p16, and SA-β-gal activity), showing a negative correlation between senescence and decidualization/implantation [19,20]. In endometriosis, recent studies have shown that defective implantation is associated with a decrease in pregnancy rates [56], demonstrating a relationship between impaired decidualization in endometriosis, and fertility. The hypothesis raised by the authors suggest that endometrial stromal cells that are senescent are irresponsive to hormone stimuli and have difficulties differentiating properly during the mesenchymal to epithelial transition; thus, senescence would impair decidualization by reducing the proliferative capacity [19]. 

Nonetheless, we have demonstrated before that endometriotic lesions express higher levels of senescence-associated proteins [13]. Now, we complement the results showing a higher concentration of ROS, the upregulation of MAPK proteins, and the higher manifestation of senescence traits on stromal cells from endometriotic lesions after stimulation with H_2_O_2_. Nevertheless, the eutopic endometrium behaves differently with lower concentrations of ROS and MAPKs when compared to the non-endometriosis endometrium and higher DNA double strands break (DSB) compared to endometriotic lesions. The differences observed in this study indicated that the eutopic endometrium functioned in a way that was opposite to that of the non-endometriosis endometrium. The human endometrium is a dynamic tissue that undergoes cyclical changes, which include shedding, proliferation, and differentiation in response to autocrine and paracrine stimuli [57]. A recent transcriptome meta-analysis reveals the higher prevalence of natural killer T cells in eutopic endometrium from women with endometriosis, insinuating the sustained stress and/or damage of the eutopic endometrium [58]. This goes in accordance with our results, which demonstrate a disturbed eutopic endometrium. Furthermore, the redox system balance is essential to trigger some of the abovementioned events since a slight increase in lipid peroxide levels occurs immediately before menstruation [59,60]. Moreover, ROS is important to maintain cellular homeostasis and shutdown or activate cellular processes such as cell cycling and self-renewal, both crucial events in endometrium [61]. Our results demonstrate that in both menstrual cycle phases there was a significant increase in DNA DSB (H2A.X) from eutopic endometrium compared to endometriotic lesions but no 8OHdG difference. Immunostained images show positive stain in both glandular and stromal cells from eutopic endometrium, whereas endometriotic lesions had a epithelial compartment prevalence. Although no comparison between cellular compartments was made in the present study, similar results have been published. High gamma H2AFX was reported in both glandular epithelial and stromal compartments of eutopic endometrium from endometriosis patients compared to non-endometriosis patients in the secretory and proliferative phases of the menstrual cycle, suggesting high DNA damage in endometrium of patients with the disease [62]. Choi and colleagues [63] have also found higher gamma H2AX in endometriosis compared to non-endometriosis but an increase in ovarian endometrioma H2AX compared to eutopic endometrium. On the contrary, the first published results on gamma H2AX in endometriosis demonstrated that in the secretory and proliferative phase, non-endometriosis endometrium had higher gamma H2AX staining than endometrium among patients without the disease, in both epithelial and stromal cells, but no comparison to endometriotic lesions was made [64]. To the best of our knowledge, we present the first study comparing eutopic endometrium and endometriotic lesions (deep infiltrating endometriosis). 

Our results are promising but should be interpreted carefully as there are limitations to this study. As presented in previous studies [13,14], p16^INK4A^ and lamin B1 proteins are more expressed in epithelial cells, and endometriotic lesions may or may not present epithelial cells, depending on the lesion type; thus, compartment-specific proteins could have their expression altered, depending on the tissue evaluated. The association between ROS and senescence traits in stromal cells must be evaluated considering different endpoints, and a quantitative analysis should be performed. Chronic and acute H_2_O_2_ sensitization may elicit different cellular responses, which should be accomplished. Ultimately, we performed a study on endometriosis-associated senescence, which indicates the influence of ROS in endometriotic stromal cells. Endometriotic lesions respond differently to ROS than eutopic and non-endometriosis endometrium cells. The answer to that remains unknown and requires further investigation. The anormal milieu in which endometriotic lesions grow could be responsible for cellular changes. Studies on whole-cell machinery, gene regulation, and perhaps on miRNA and the vesicles responsible for activating or depleting specific senescence-trigger proteins may be required. Understanding the mechanism underlying the long-lasting persistence of endometriotic lesions will build a consistent base to unroll appropriate drug therapy for the definitive treatment of the disease, which still remains incurable. Notwithstanding, the current treatments available are either surgery or hormonal/anti-inflammatory drugs, all with disease remission reported. 

## 4. Materials and Methods

### 4.1. Patients and Samples

This study was part of the Women’s Health Program of Hospital Israelita Albert Einstein (HIAE), Sao Paulo, Brazil. Fifty-one patients were selected between September 2017 and September 2019, examined by a single gynecologist surgeon (SP), and divided into two groups: endometriosis (n = 33 patients) and non-endometriosis (n = 20 patients). Biopsies of the eutopic endometrium (non-endometriosis = eutopic endometrium of a patient without endometriosis, and eutopic = eutopic endometrium of a patient with endometriosis) and deep infiltrating endometriosis (endometriotic lesion) were obtained during surgery. The patients involved were part of our previously described sample cohort [13], who provided proliferative and secretory phase samples.

A written informed consent form was obtained from all patients. The study was approved by the Human Research Committee of Hospital Albert Einstein (CAAE: 56229916.9.0000.0071; Sao Paulo, Brazil), affiliated with the Ethics Committee of the Brazilian Ministry of Health (CONEP). The diagnosis of endometriosis was made after visualizing the lesions during surgery, which was subsequently confirmed via histopathological analysis at the HIAE Pathological-Anatomical Laboratory. In patients without endometriosis, the absence of endometriosis was confirmed during surgery. 

The following inclusion criteria were applied to both groups: age between 18 and 50 years; eumenorrheic menstrual cycles with interval of 24–35 days [65]; and no hormonal therapy, including gonadotropin-releasing hormone analogs, progestins, and oral hormonal contraceptives in the six months before surgery. Patients who met one or more of the following criteria in the three months prior to laparoscopy were excluded from the study: status as smoker; the presence of hydrosalpinx and endometrial polyps; and a diagnosis of diabetes mellitus or other endocrinopathies, cardiovascular disease, dyslipidemia, systemic lupus erythematosus, or other rheumatological or oncological diseases. 

### 4.2. Experimental Design

The number of patients distributed throughout the study is presented in Table 1. This work was based on an exploratory cross-sectional pilot study. The number of patients for the immunohistochemistry experiments was estimated to yield a difference of 7.88%, a standard deviation of 5.6%, a significance level of 5%, and a power of 85%. For the other evaluations and experiments, the remaining biological samples were used for the analyses, making this a prospective exploratory study. Patients without endometriosis provided samples of eutopic endometrial biopsies (9 proliferative and 9 secretory) for OS and MAPK quantification. Two non-endometriosis patients were donors of endometrium biopsies for cell culture experiments. Patients with endometriosis collectively provided 24 eutopic endometrium (13 proliferative and 11 secretory) and 27 endometriotic lesions (15 proliferative and 12 secretory) that were distributed among OS and MAPK experiments. Not all experiments had the same number of tissues. Six patients provided eutopic endometrium and endometriotic lesions for cell cultures in vitro experiments. 

Patients underwent video laparoscopy for the resection of endometriotic lesions and the evaluation of pelvic pain and myomectomy for intramural fibroids only. Deep retrocervical samples of endometriosis lesions were obtained for this study. For both groups, endometrium was collected using a Recamier Weldon curette shortly after the patient was positioned for surgery. All of the collected tissues used for in vivo studies were stored at −80 °C. After surgical removal, the tissues collected for cell culture were immersed in a culture medium and subsequently processed in the experimental laboratory. 

We first aimed to investigate the in vivo levels of OS biomarkers (H2A.X, deoxyguanosine (8OHdG), the carbonyl group, ferric-orange xylenol (FOX), malondialdehyde (MDA), reduced glutathione (GSH), and oxidized glutathione (GSSG)) by absorbance assays, and MAPK phosphorylated protein using Luminex MagPix immunological assay, to understand the eutopic endometrium and endometriotic lesion microenvironment. Then, using in vitro assays, we investigated primary stromal cells from patient samples with and without endometriosis exposed to sublethal oxidative stress. We aimed to evaluate the H_2_O_2_-treatement on senescence markers. 

The proliferative and secretory phases were divided according to the patient-reported onset of the menstrual cycle. The proliferative phase was considered 0–15 days after the first day of the last menstrual period, and the secretory phase was considered 16–35 days after the first day of the last menstrual period. 

### 4.3. Preparation of Tissue Homogenate for OS and MAPK Analysis

Non-endometriosis endometrium, eutopic endometrium, and endometriotic lesion samples were weighed (between 18 and 22 mg) and placed in an Eppendorf tube. After the addition of phosphate buffer saline, the tissue was perforated with sterile scissors, sonicated in the Vibra-Cell™ ultrasonic liquid processor (SONICS) at an amplitude of 20%, and centrifuged at 4000 rpm at 4 °C for 10 min. The supernatant was separated into two parts for analysis.

### 4.4. Protein Quantitation

Protein concentration was determined using the Pierce™ bicinchoninic acid protein assay kit (Thermo Fisher Scientific, Waltham, MA, USA). Measurements were made using the Spectramax instrument (Beckman Coulter, Brea, CA, USA, DTX880 multimode detector) at 540 nm. Results were expressed as micrograms per microliter or total grams per total sample volume.

### 4.5. ROS Formation 

The total hydroperoxide (FOX) content was determined as described previously [66]. The ROS content was measured in the three tissues collected (non-endometriosis endometrium, eutopic endometrium, and endometriotic lesion). The FOX assay system is based on the oxidation of Fe^+2^ (ferrous ions) to Fe^+3^ (ferric ions) by different types of peroxides presents in the samples under investigation. In the presence of xylenol orange, a blue-violet colored complex (xylenol orange iron) is formed, the absorbance of which can be measured at 570 nm [67]. In this method, a substance prepared on the day of measurement (the FOX reagent) was used. Solution 1 comprised xylenol orange (Sigma–Aldrich, Saint Louis, MO, USA; 52097-1G) and butylated hydroxytoluene (BHT) (Sigma Aldrich; 47168) in methanol (Sigma–Aldrich, Saint Louis, MO, USA; 322415-1L). Solution 2 comprised 250 mM ferrous sulfate (Sigma–Aldrich, Saint Louis, MO, USA; F1543) in sulfuric acid (Sigma–Aldrich, Saint Louis, MO, USA; 339741-100ML). The two solutions were mixed in a 9:1 ratio at the time of reading. For analysis, tissue homogenate, water (for the blank), and the solution were added and centrifuged at 3500 rpm between 22 and 24 °C for 10 min, and the absorbance of the supernatant was measured at 560 nm. The curve was plotted using H_2_O_2_ (Sigma–Aldrich, Saint Louis, MO, USA; 95299-1L) at concentrations ranging from 10–200 µM. The result was expressed as nmoL/g protein.

### 4.6. Detection of Oxidative Damage to Lipids 

This analysis was performed as described previously [68], with some modifications. The tissue homogenate was transferred to an Eppendorf tube, to which 10 mM 1-methylphenylindole (Sigma–Aldrich, Saint Louis, MO, USA; 404888-10G) in acetonitrile (Sigma–Aldrich, Saint Louis, MO, USA; 34851-1L), methanol (2:1, *v*/*v*), and 37% hydrochloric acid (Sigma–Aldrich, Saint Louis, MO, USA; 320331-500ML) were added. The Eppendorf tubes were shaken and incubated in a water bath at 45 °C for 1–2 h. The samples were then cooled on ice and centrifuged at 4000 rpm between 22 and 24 °C for 10 min. The absorbance of the supernatant was measured at 586 nm. The MDA concentration was calculated by comparing it with the curve generated using hydrolyzed 1,1,3,3-tetramethoxypropane (Sigma–Aldrich, Saint Louis, MO, USA; 108383-100ML).

### 4.7. Detection of Oxidative Protein Damage (Carbonyl Group)

The samples of tissue homogenate were separated and mixed with trichloroacetic acid (Sigma–Aldrich, Saint Louis, MO, USA; T6399-5G), shaken, and centrifuged at 3500 rpm between 22 and 24 °C for 10 min. The supernatant was discarded, and 2,4-dinitrophenylhydrazine (Sigma–Aldrich, Saint Louis, MO, USA; D199303-25G) was added. The samples were incubated at 37 °C for 2 h in the dark and shaken repeatedly. After incubation, the samples were washed with ethanol/ethyl acetate mixture (Sigma–Aldrich, Saint Louis, MO, USA; 270989-100ML) (*v:v*), vortexed, and centrifuged at 3500 rpm at 4 °C. After washing, the pellets were dissolved in 1 M sodium hydroxide (Sigma–Aldrich, Saint Louis, MO, USA; S8045-500G) in a water bath at 34 °C. The samples were placed in a 96-well straight bottom plate (Thermo Fisher Scientific, Waltham, MA, USA) and read at 360−390 nm. The following equation was used to calculate the carbonyl concentration [69]: A = ε × b × C, where A = absorbance; ɛ = molar extinction coefficient of the carbonyl; b = optical distance (0.5 cm for the 200 µL placed in the plate); and C = the expressed concentration.

### 4.8. Detection of Oxidative DNA Damage (8OHdG)

The amount of 8OHdG was quantified and estimated using enzyme-linked immunosorbent assay (ELISA) (Stressgen^®^ DNA Damage ELISA Kit, Ann Arbor, MI, USA), according to the manufacturer’s recommendations. The calibration curve was plotted using known concentrations of 8OHdG ranging from 0.94–10 ng/mL. DNA and the standards were added to each well in a plate previously sensitized with anti-8OHdG antibody (anti-8-hydroxyguanosine antibody (ab10802)). After incubation between 22 and 24 °C, the plate was washed with buffer using the automatic microplate washer (Bio-Rad, Hercules, CA, USA). TMB substrate was added to each well and incubated in the dark, and finally the stop solution (STOP solution, Stressgen^®^ DNA Damage ELISA Kit, Ann Arbor, MI, USA) was added. A yellow color was detected, which was inversely proportional to the concentration of 8OHdG. Absorbance was measured at 450 nm using the instrument and Glow Max Discovery software (Promega, Medison, WI, USA).

### 4.9. Immunohistochemistry

Immunohistochemistry was performed following the protocol of [70] with modifications described in [13]. Briefly, staining was performed using a 1:1000 dilution of mouse monoclonal antibody against H2A.X (phospho S139, phosphorylated variant of histone H2A) (ab22551; Abcam, Cambridge, UK). Photomicrography was performed using an IX-51 microscope (Zeiss, Oberkochen Germany). Immunohistochemistry was performed using a BenchMark ULTRA IHC/ISH marking platform (Ventana Medical Systems, Oro Valley, AZ, USA). For quantitative analysis, the histometric method, a semiquantitative subjective scoring system, was used to assess the protein-positive areas [71,72]. Positive staining analysis was validated using the method described previously [73], which indicated high correlation between the results of objective computerized image analysis and subjective semiquantitative manual visualization. For histometric analysis, the IX51 inverted microscope was used to visualize, photograph, and quantify the images using Cell Sens Dimension software (Olympus, Center Valley, PA, USA).

### 4.10. Antioxidant Response to Intracellular Accumulation of ROS 

The concentrations of intracellular antioxidant enzymes were measured using GSH and GSSG absorption assays. Briefly, the tissue homogenate was processed as described previously [74], with thiol groups reacting with dithionitrobenzoic acid (DTNB) (Sigma–Aldrich, Saint Louis, MO, USA; D8130-500MG) to form an anion with a maximum peak at 412 nm (e412 = 13, 600 M^−1^ cm^−1^). An aliquot of the tissue homogenate was mixed with Tris-EDTA buffer (25 nmoL/L Tris base, 20 mmoL/L EDTA, pH 8.2; Sigma Aldrich; T9285-100ML), and the absorbance was measured at 412 nm. The DTNB stock solution (10 nmoL/L in absolute ethanol; Sigma–Aldrich, Saint Louis, MO, USA; 32205-1L) was added to this solution, followed by incubation between 22 and 24 °C and measurement of absorbance at 412 nm, with DTNB as the blank [67]. The concentration of sulfhydryl groups was calculated using GSH, and the results were expressed in nanomoles per gram of protein.

### 4.11. MAPK Pathway Detection

The homogenized tissues were analyzed for key proteins of the MAPK pathway using the multiplex immunoassay and the MAPK/SAPK signaling 10-plex magnetic bead kit in a 96-well plate (cat # 48-660MAG, Merck Millipore^®^, Burlington, MA, USA). The measurement was performed using a Millipex Map instrument (Merck Millipore^®^, Burlington, MA USA). This panel comprised the following phosphorylated proteins: ERK1/2, JNK, P38, ATF2, c-Jun, p53, STAT1, MEk1, MSK1, and HSP27. The samples, standard curve, and blank were added to the 96-well plate, according to the manufacturer’s instructions. The assay buffer and magnetic beads (antibodies) were added to each well, and the plate was incubated for 14–16 h at 4 °C. The antibody-detection solution was added on the second day. The plate was incubated with the detection antibodies and then with streptavidin-phycoerythrin (SAPE) for detection using the Millipex Map instrument. The amplification buffer was added to the SAPE solution. The solution was removed, and 100 µL of assay buffer was added for plate reading. The reading was performed on the Luminex^®^200™ using the MAGPIX^®^ software for reading and data analysis.

### 4.12. Stromal Cell Culture 

The primary cultures of the non-endometriosis and endometriosis endometrial stromal cells and endometriotic lesion stromal cells were performed as described previously [75]. Briefly, the tissues were digested for 40 min at 37 °C with 0.5% collagenase type IV (Sigma–Aldrich, Saint Louis, MO, USA). The cells were isolated in Dulbecco’s modified Eagle’s medium (DMEM, Gibco Laboratories, Grand Island, NY, USA). A 250-mm sieve (Sigma–Aldrich, Saint Louis, MO, USA) was used to filter the cell suspensions. A second sieve with 35-mm mesh was used to retain the clustered cells, such as epithelial cells. The flow-through with the single cells was seeded in flasks. Culture and expansion were performed in DMEM supplemented with 15% fetal bovine serum and 1% antibiotic/antimycotic solution (100×) (Gibco Laboratories, Grand Island, NY, USA) at 37 °C in a 5% CO_2_ incubator. After six passages, the absence of epithelial cell contamination was assessed by immunostaining the cells with anti-vimentin as a specific marker of stromal cells and anti-E-cadherin, a specific marker of epithelial cells.

### 4.13. Experimental Treatments 

Once the purity and specificity of the stromal cells were assessed, cells cultured until passage 5 were once again plated in a 96-well plate at a concentration of 10,000 cells per well (now passage 6). The cells were kept in culture for adhesion for 24 h, following which DMEM (0.0 mM for control and 0.25 mM H_2_O_2_) was added. The same cells, plated in different wells, received both concentrations of H_2_O_2_, making it possible to compare the performance of the same cells in both settings (0.0 mM and 0.25 mM H_2_O_2_). The cells were allowed to respond for 24 h, followed by immunofluorescence analysis.

### 4.14. Immunofluorescence of Cultured Stromal Cells 

The cultured cells mentioned above were fixed with 4% paraformaldehyde in phosphate buffered saline (PBS), followed by cellular permeabilization with 0.01% Triton-X 100 and 30 min of incubation between 22 and 24 °C in a blocking solution comprising PBS with 1% bovine serum albumin. Subsequently, the cells were incubated for 14–16 h with primary antibodies against vimentin (1:200, catalog no. Sc-373717; Santa Cruz Biotechnology, Dallas, TX, USA), E-cadherin (1:200, Dako Agilient, Santa Clara, CA, USA), p16^ink4a^ (1:100, rabbit monoclonal (EPR1473) to CDKN2A/p16INK4a tag; Abcam, Cambridge, UK), and lamin B1 (1:50, H90, catalog no. Sc-20682; Santa Cruz Biotechnology), in separate wells. The Alexa 488-conjugated secondary antibody (Cell Signaling, Beverly, MA, USA) and Alexa Fluor 694-conjugated antibody (Jackson Immunoresearch Laboratories, West Grove, PA, USA) were used as secondary antibodies (1:400 dilution). Nuclear staining was performed using Vectashield medium containing DAPI (Vector Laboratories, Burlingame, CA, USA). Cells were also incubated with Cell Trace Violet Kit—cell proliferation (C34557—Thermo Fisher Scientific, Waltham, MA USA) for morphology characterization. Cell images were acquired with an LSM710 confocal microscope (Zeiss, Jena, Germany). HeLa cells were used as positive controls for p16^ink4a^ and lamin B1 staining.

### 4.15. Quantification of the Positive Staining of Stromal Cells

The Zen Blue program was used to quantify protein expression. This program quantifies the pixels emitted in each channel. Two-channel readings, at 405 nm (DAPI) and 488 nm (Alexa Fluor 488 used as the secondary antibody for p16^Ink4a^ and lamin B1), were selected. Each reading was taken proportional to the two channels. The quantified pixels from one channel (p16^Ink4a^/DAPI and lamin B1/DAPI = 488 nm/405 nm) were divided by those from the other to equalize the expression of p16^Ink4a^ and lamin B1 according to the number of cells present in each image. Thus, images with few or many cells showed proportional expression of p16^Ink4a^ and lamin B1.

### 4.16. β Galactosidase Assay

Senescence-associated β-galactosidase (SA-β-gal) activity was detected using the senescence detection kit (Ab65351; Abcam, Cambridge, AX, UK) according to the manufacturer’s instructions. After H_2_O_2_ treatment, the stromal cells were fixed for 10 min between 22 and 24 °C in the dark and washed once with the buffer provided in the kit. The labeling solution containing the substrate (X-gal) was prepared daily according to the manufacturer’s instructions, and the pH was adjusted. The labeling solution was added to the cells, and the culture plate was sealed with parafilm and incubated for 4 h at 37 °C in the dark. The SA-β-gal detection solution was removed, and the stromal cells were washed with buffer. The SA-β-gal-positive blue cells were recorded under an inverted light microscope (Thermo Fisher Scientific, Waltham, MA USA) equipped with a digital camera, and five randomly selected fields were counted to determine the percentage of β-gal-positive cells. 

### 4.17. Data Analysis and Interpretation 

Quantitative variables were described using the median and interquartile range as they did not have normal distribution (according to the Shapiro–Wilk test) [76]. Shapiro–Wilk tests were used to assess the distribution of all quantitative variables. Generalized linear mixed models with gamma or normal distribution were fitted considering the variables groups (non-endometriosis, eutopic endometrium, and lesion) and menstrual cycle phase (secretory and proliferative). Results were presented as estimated means and 95% confidence intervals (CI) or mean ratios (MR) between groups and 95% CI for comparisons of interest. These models accounted for dependence between measurements of the same individual and within the same group, as we considered 16 replicates for H2A.X assessment. For ROS and MAPK, the model analyzed the dependence between measurements within the same group and more broadly within the non-endometriosis and endometriosis groups (eutopic endometrium and lesion) [77]. A significance level of 5% was considered. The statistical package R (R Core Team 2017) was used.

## 5. Conclusions

The current work presents, in a concise manner, the consistent ROS presence in endometriosis, the dysregulation of MAPK expression, and the deleterious impact of ROS on the expression of senescence markers (which was observed in stromal cells from endometriotic tissues). The results demonstrated in this study contribute to the understanding of the cell senescence path in endometriotic lesions and provide insights into the long-term persistence of the disease. The presence of senescent cells in endometriosis, upregulated by consistent ROS and the dysregulation of MAPK, could shift the way we currently treat patients. Several studies have reported that pharmaceutical inhibitors of MAPK with other key dysregulated pathways are used as an attractive target for therapy in cancer, and we should explore this approach in endometriosis. Knowing that there is an increase in ERK1/2, p38, and JNK in endometriotic lesions, at least in the proliferative phase, and that this could enhance cells to achieve senescence traits and proliferate, the administration of synthetic MAPK signaling inhibitors, such as the Raf inhibitor or MEK1/2 inhibitor, could suppress their function and culminate in a decrease in lesion size by making endometriotic lesions susceptible to apoptosis. 

## 6. Summary Hypothesis Schema

**Figure 6:**
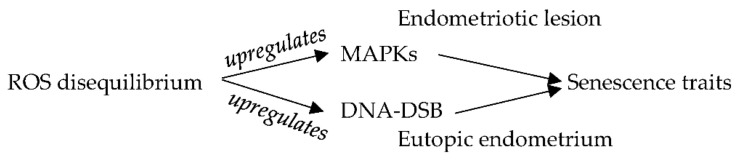
Summary Hypothesis Schema.

The hypothesis (Figure 6) of our work is based on ROS disequilibrium altering cellular behavior to develop senescence traits in both endometriotic lesions and eutopic endometrium. 

## Figures and Tables

**Table 1:** Experimental samples, and phases of the menstrual cycle.

	Endometriosis (n = 33)				Non-Endometriosis (n = 20)	
	Proliferative (n = 15)		Secretory (n = 12)		Proliferative (n = 9)	Secretory (n = 9)
	Lesion	Endometrium	Lesion	Endometrium	Endometrium	Endometrium
Assays						
H2A.X	15	13	12	11	9	9
FOX, MDA, Carbonyl, GSG, GSSG	8	8	12	11	7	9
MAPK	8	8	12	11	7	9
8OhdG	7	2	6	7	6	7
Cell culture	6				2	

Note. MDA: malondialdehyde; FOX: ferric-orange xylenol, GSH: reduced glutathione; GSSG: oxidized glutathione; H2A.X: Histone H2A variant; MAPK: mitogen-activated protein kinase; and 8OhdG: 8 deoxyguanosine.

## Data Availability

The data that support the findings of this study are available on reasonable request from the corresponding author (HM). The data are not publicly available as they contain information that could compromise the privacy of the participants.

## Supplementary Materials

The following supporting information can be downloaded at: https://www.mdpi.com/article/10.3390/ijms24020914/s1.

## Abbreviations

ROS, reactive oxygen species; OS, oxidative stress; SASP, senescence-associated secretor phenotype; 8OHdG, deoxyguanosine; FOX, ferric-orange xylenol; MDA, malondialdehyde; GSH, reduced glutathione; GSSG, oxidized glutathione; and SA-β-gal, senescence-associated β-galactosidase.
