# Supplementary material for: Higher Oxidative Stress in Endometriotic Lesions Upregulates Senescence-Associated p16ink4a and β-Galactosidase in Stromal Cells
Source: Int J Mol Sci. 2023 Jan 4;24(2):914. doi: 10.3390/ijms24020914 (PMC9860681; doi:10.3390/ijms24020914)
Supplement: Supplementary file 1 [file ijms-24-00914-s001.zip › ijms-2070616-supplementary.pdf]

Supplementary Material

Supplementary Figure S1

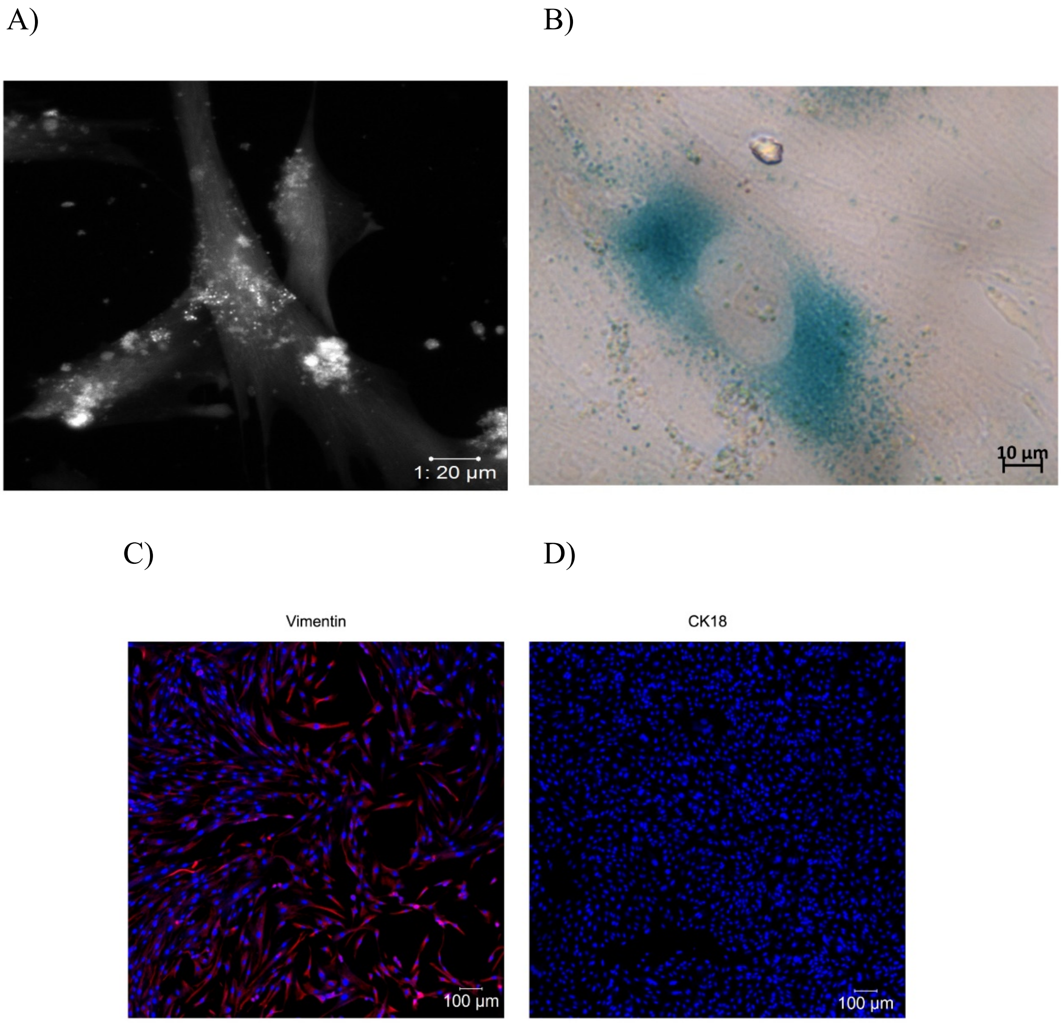

Supplementary Figure S1. Morphological features of the endometriotic stromal cells. Representative photomicrographs of the endometriotic stromal cells at the 6th passage in culture, showing typical elongated and spindle-like morphology. The cells stained positive for (A) Cell trace violet (B) β-galactosidase, and (C) vimentin; and negative for (D) CK18 in immunofluorescence analysis. The nuclei were stained with DAPI.

Supplementary Table S1. $p16^{ink4}$ and Lamin B1 expression ratio in stromal cell

	Ratio 0.25mM/0mM	
Group	Lamin b1	$p16^{ink4}$
Non-endometriosis	0.22	1.29
Non-endometriosis	0.38	1.30
Eutopic Endometrium	3.05	8.38
Eutopic Endometrium	1.05	1.28
Eutopic Endometrium	1.62	1.53
Eutopic Endometrium	1.60	4.43
Eutopic Endometrium	0.84	0.40
Eutopic Endometrium	8.47	3.29
Endometriotic Lesion	0.74	2.78
Endometriotic Lesion	0.97	0.47
Endometriotic Lesion	0.72	4.75
Endometriotic Lesion	1.43	2.59
Endometriotic Lesion	-	0.66
Endometriotic Lesion	-	1.37
